# Association between CTL Precursor Frequency to HLA-C Mismatches and HLA-C Antigen Cell Surface Expression

**DOI:** 10.3389/fimmu.2014.00547

**Published:** 2014-10-27

**Authors:** Moshe Israeli, Dave L. Roelen, Mary Carrington, Effie Wang Petersdorf, Frans H. J. Claas, Geert W. Haasnoot, Machteld Oudshoorn

**Affiliations:** ^1^Department of Immunohematology and Blood Transfusion, Leiden University Medical Center, Leiden, Netherlands; ^2^Tissue Typing Laboratory, Rabin Medical Center, Petach Tikva, Israel; ^3^Cancer and Inflammation Program, Laboratory of Experimental Immunology, Frederick National Laboratory for Cancer Research, Leidos Biomedical Research, Inc., Frederick, MD, USA; ^4^Ragon Institute of Massachusetts General Hospital, Massachusetts Institute of Technology and Harvard University, Boston, MA, USA; ^5^Clinical Research Division, Fred Hutchinson Cancer Research Center, Seattle, WA, USA; ^6^Europdonor Foundation, Leiden, Netherlands

**Keywords:** cytotoxic T-cell precursor frequency, HLA-C, cell surface expression, allo-reactivity, CTLp assay

## Abstract

Previous studies showed the relevance of the cytotoxic T-cell precursor (CTLp) frequency assay for prediction of the outcome of HLA mismatched hematopoietic cell transplantation (HCT). Recently, it has been shown that HLA-C cell surface expression is correlated with virus specific cytotoxic T-cell responses and viremia control in HIV patients. The aim of the current study was to investigate the association between HLA-C antigen expression and the CTLp frequency to the mismatched HLA-C antigen. In total 115 recipient–donor pairs, for whom a successful CTLp assay was performed, were evaluated for this pilot study. All donor–recipient pairs were matched at 9/10 alleles with a single mismatch at the HLA-C locus. Antigen expression level of the mismatched HLA-C allele for each recipient and donor was based on the mean fluorescence intensity (MFI) values as described by Apps et al. (1). The cell surface expression of recipient’s mismatched HLA-C antigen was significantly lower among CTLp negative (*n* = 59) compared to CTLp positive (*n* = 56) pairs (154 and 193 MFI units, respectively, *p* = 0.0031). This difference was more pronounced in donor–recipient pairs that were mismatched for amino-acid residue-116 located in the groove of the HLA-C antigen, suggesting that the importance of peptide binding in the allo-recognition. Furthermore, in the particular case of low expression of the recipient mismatched HLA-C antigen (MFI < 115), CTLp reactivity depended on HLA-C expression level in the donor, the median MFI of donor’s mismatched HLA-C antigen was 114 in CTLp negative cases (*n* = 26), while in CTLp positive cases (*n* = 15) the median MFI of donor’s HLA-C antigen was 193 (*p* = 0.0093). We conclude that the expression level of the donor and recipient mismatched HLA-C antigens affect CTLp outcome. HLA-C antigen expression levels in combination with the CTLp assay may prove useful for the prediction of the clinical outcome of HLA-C mismatched HCT.

## Introduction

Matching of HLA-C alleles between donor and recipient is associated with favorable outcome in hematopoietic cell transplantation (HCT) (2) and in cord blood transplantation (3). However, recent data show that the immunogenicity of HLA Class I mismatches may vary. Some Class I mismatches lead to a strong allo-immune response associated with severe clinical complications such as graft versus host disease (GvHD), whereas others are associated with better clinical results due to lack of a detrimental allo-immune response (4, 5). Previous studies showed the relevance of the cytotoxic T-cell precursor (CTLp) frequency assay for predicting outcome of HLA mismatched HCT (6). Patient survival is significantly better if no cytotoxic T-cells (CTLs) are present in the donor versus the recipient’s HLA mismatched antigen, compared to a donor–recipient combination with a positive CTLp test (7). Several approaches for characterizing differential allo-reactivity amongst HLA mismatches have been suggested, such as compatibility in killer-immunoglobulin receptors (KIR) or at specific amino-acid residues. However, none of these concepts has been able to provide a coherent reliable prediction of allo-reactivity and its clinical consequence in HCT.

Recently, it has been shown that HLA-C cell surface expression is correlated with virus specific cytotoxic T-cell responses and viremia control in HIV patients (1, 8–10) as well as in protection against Crohn disease (CD) (1, 10). Apps et al. (1) have described a method for coherent quantitative evaluation of HLA-C cell surface expression by measurement of the mean fluorescence intensity (MFI) for every serologically defined HLA-C antigen. The aim of the present study was to investigate the possible association between HLA-C antigen cell surface expression and the CTLp assay outcome. As the CTLp assay is technically very difficult, these studies may lead to an alternative and easier approach to predict T-cell mediated allo-reactivity, thus, facilitating donor selection for HCT.

## Materials and Methods

### Patients and donors

We studied 115 recipient–donor pairs registered by the Europdonor Foundation, for whom a successful CTLp assay was performed (114 unrelated, 1 related). Patients were adults and children referred for HCT in one of three Dutch transplant centers: Leiden University Medical Center (LUMC), Erasmus Medical Center – Daniel den Hoed, and University Medical Centre Utrecht (UMCU) – Wilhelmina Children’s Hospital. Patients were diagnosed with either malignant or non-malignant diseases. The unrelated donors were from Europdonor Foundation or from international donor registries. All donor–recipient pairs were matched at 9/10 alleles at allele-level resolution with a single allele or antigen mismatch at the HLA-C locus. All testings were performed as part of routine clinical work-up in preparation for donor selection for allogeneic transplantation. The data for this study were collected retrospectively and anonymously from laboratory records.

### HLA genotyping and CTLp assay

All donors and patients were typed for HLA-A, -B, -C, -DRB1, and -DQB1 at allelic resolution as described previously (11). In brief, polymerase chain reaction sequence specific primer and sequence based typing were used. The CTLp assays were performed as described by Zhang et al. (12), with minor modifications as described by Oudshoorn et al. (11). CTLp assays were performed in the graft versus host (GvH) direction: donor cells were used as responder cells and patient cells as stimulator and target cells. A negative CTLp result was defined as ≤1 recipient-specific CTL per 10^6^ peripheral blood lymphocytes.

### HLA-C match characteristics

Antigen expression level of HLA-C alleles was based on the MFI values for each Cw serologic equivalent as described by Apps et al. (1). Alignment of amino-acid sequences of donor and recipient HLA-C alleles for determination of position-116 matching was performed using the Immuno Polymorphism Database website of the European Bioinformatics Institute (13). KIR-ligand compatibility prediction was based on donor and recipient HLA-B and HLA-C allelic resolution typing using the KIR-ligand calculator provided in the Immuno Polymorphism Database website of the European Bioinformatics Institute (13). As described by Ruggeri et al. (14), KIR-ligand compatibility was defined as presence in recipients of donor Class I allele antigen groups recognized by KIRs, and incompatibility was defined as absence in the recipient of donor Class I antigen groups recognized by KIRs.

### Statistical analysis

Comparison of independent MFI values between two groups was performed using the Mann–Whitney *U* (MWU) test, as this parameter is associated with HLA-C allele type and therefore not normally distributed.

For the univariate and multivariate logistic regression analyses, C antigen cell surface expression levels of the mismatched HLA-C allele, as expressed in predicted MFI units based on previous determination of mean expression of each allele, were first calculated as a discrete numerical variable and in addition were dichotomized into a binary variable of high- (MFI > 200) or low- (MFI ≤ 115) expression levels. In regard to the discrete parameters, the change in OR shown for HLA-C expression represents an increase of 50 MFI expression units, taking the OR determined for an increase of a single unit and converting it by following: e^(ln(OR)*50)^. Recipient and donor mismatched C allele MFI levels were combined together for the univariate logistic regression analysis in the following manner: (1) A discrete numerical interaction variable calculated by multiplication of the two discrete numerical variables. (2) A binary variable: category 0 is of donor–recipient pairs where both HLA-C mismatched alleles are low (MFI ≤ 115) and the category 1 is of pairs where at least one of the HLA-C mismatched alleles is high (MFI > 200). Multivariate logistic regression analysis, based on all parameters that were found to be statistically significant in the univariate analysis, was performed by stepwise elimination based on the Wald statistic. Statistical significance was declared at *p* < 0.05. All analyses were performed using SPSS 20.0.0 (IBM SPSS Corporation, Chicago, IL, USA).

## Results

### Association of recipient and donor C antigen expression with CTLp outcome

**Figure 1:**
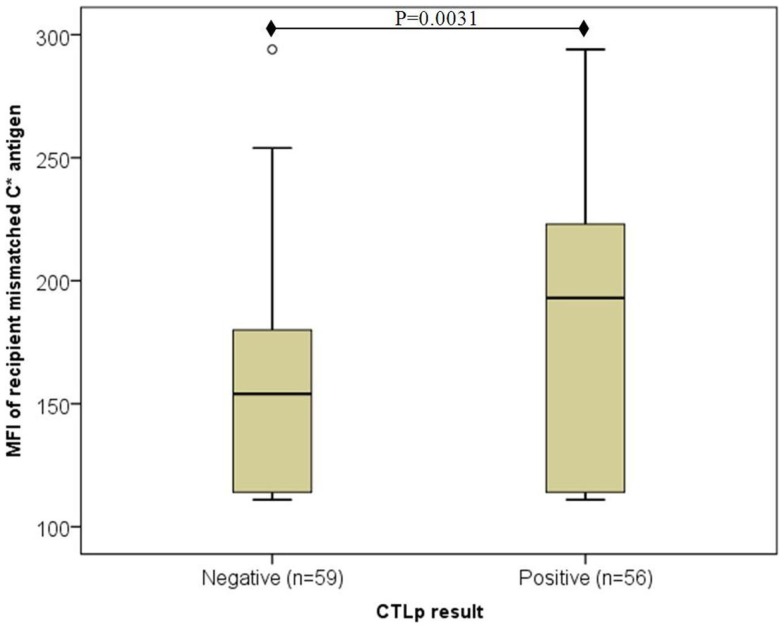
**Association of recipient mismatched C antigen cell surface expression level with CTLp result**. Median MFI of recipient’s mismatched HLA-C antigen cell surface expression was significantly lower among CTLp negative compared to CTLp positive pairs (*p* = 0.0031, MWU test). Horizontal line represents median MFI, box plot represents MFI values between the 25th and 75th percentiles, and thin whiskers represent range.

Overall, median MFI of recipient’s mismatched HLA-C antigen expression was significantly lower among CTLp negative (*n* = 59) compared to CTLp positive (*n* = 56) pairs (154 and 193, respectively, *p* = 0.0031, MWU test) (see Figure 1). The MFI level of the donor’s mismatched HLA-C antigen was similar in CTLp negative or positive pairs (164 and 170, respectively, *p* = 0.081, MWU test).

#### Particular cases of low-recipient HLA-C expression

**Figure 2:**
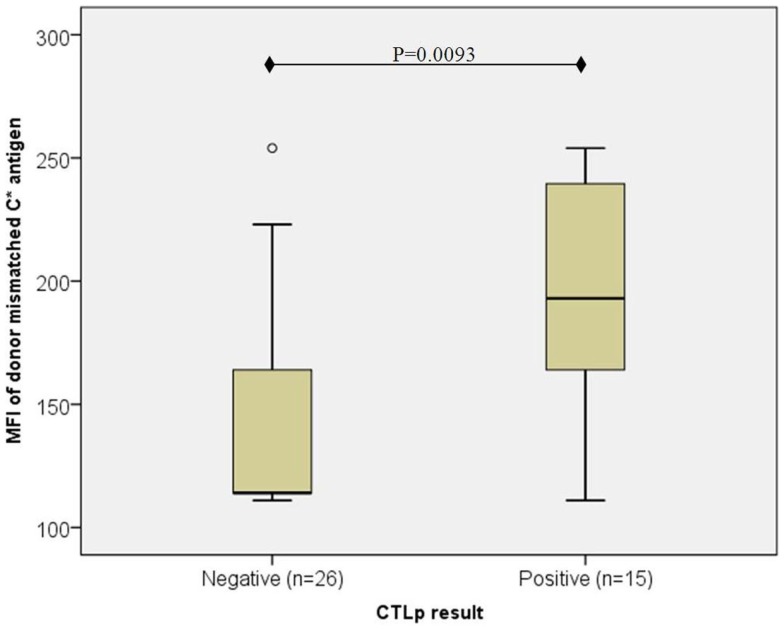
**Association of donor mismatched C antigen cell surface expression with CTLp result in cases where the recipient’s antigen is of low expression ( <115)**. The median MFI of the donor’s mismatched HLA-C cell surface expression level was lower in CTLp negative cases than in CTLp positive cases (*p* = 0.0093, MWU test) among pairs where the recipient’s HLA-C expression level of the mismatched allele was low (MFI < 115). Horizontal line represents median MFI, box plot represents MFI values between the 25th and 75th percentiles, and thin whiskers represent range.

However, in the particular cases where recipient HLA-C expression was low (C*07 or C*03; MFI < 115), CTLp reactivity appeared to depend on the HLA-C expression level in the donor (as depicted in Figure 2). In CTLp negative cases (*n* = 26), the median MFI of donor’s mismatched HLA-C antigen was 114, while in CTLp positive cases (*n* = 15), the median MFI of donor’s HLA-C antigen was 193 (*p* = 0.0093, MWU test).

#### Particular cases of high-recipient HLA-C expression

On the other hand, in particular, cases where the recipient HLA-C was high (C*06, C*18, C*01, and C*14; MFI ≥ 225) no correlation between the donor’s HLA-C antigen expression and the CTLp result was observed (data not shown).

### Donor–recipient HLA-C match characteristics

Next, the association between HLA-C expression and CTLp allo-reactivity was analyzed taking into consideration previously described HLA-C match characteristics, which affect either CTLp or clinical outcome:

#### HLA-C*03:03–C*03:04 as a permissible mismatch

Previous publications (5, 11) have identified the combination of C*03:03–C*03:04 as a permissible mismatch that is better tolerated than other HLA mismatches. This is likely due to the fact that the amino-acid substitution in residue 91 (located in a loop connecting the α1 and α2 domains) is located at a place on the HLA-C molecule that is not recognized by the T-cell receptor (TCR). In our study cohort, nine pairs presented a mismatch of C*03:03–C*03:04 alleles between the donor and recipient. All of these nine pairs resulted in a negative CTLp. When excluding these pairs from the overall analysis, the difference between the medians of CTLp negative (*n* = 50) and CTLp positive (*n* = 56) remains significant (159 and 193, respectively, *p* = 0.0173, MWU test).

#### Position-116 match

Several publications have indicated that matching the amino-acid at position-116 in the HLA-C protein confers protection against the risk for acute GvHD (4, 15). In the current study cohort, we did not find a significant association between matching at position-116 and CTLp result. Positive CTLp allo-reactivity was detected in 17 of the 37 position-116 matched pairs (46%), which is comparable to 39 of the 78 position-116 mismatched pairs (50%) (*p* = 0.7, Fisher’s exact test).

**Figure 3:**
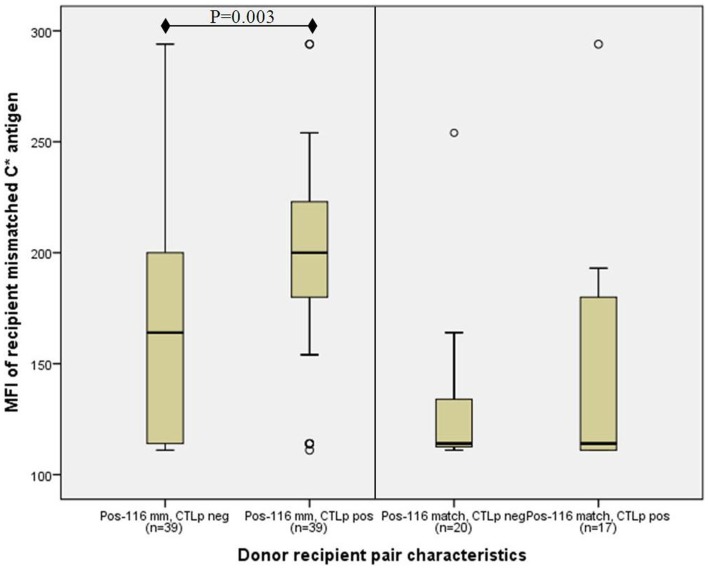
**Association of recipient C antigen cell surface expression level with CTLp result is only significant in donor–recipient pairs that are mismatched at amino-acid position-116**. Median recipient mismatched HLA-C MFI among CTLp positive pairs was significantly higher than in CTLp negative pairs among pairs that were mismatched at position-116 (*p* = 0.003, MWU test; left panel). No significant MFI level difference between CTLp positive and negative pairs was identified in cases where position-116 was matched (right panel). Horizontal line represents median MFI, box plot represents MFI values between the 25th and 75th percentiles, and thin whiskers represent range.

However, the median MFI of the recipient’s mismatched HLA-C antigen where position-116 was mismatched (187, *n* = 78) was significantly higher than in cases in which position-116 was matched (114, *n* = 37) (*p* < 0.001, MWU test). Furthermore, the association between high-HLA-C cell surface antigen expression of the recipient’s mismatched allele and a positive CTLp was augmented in position-116 mismatched pairs, where the median recipient MFI among CTLp positive pairs (200, *n* = 39) was significantly higher than in CTLp negative pairs (164, *n* = 39) (*p* = 0.0030, MWU test; Figure 3). No significant difference in MFI levels between CTLp positive and negative pairs was identified among pairs that were matched in position-116 (median 114 in both groups, *p* = 0.411, MWU test).

#### NK cell allo-reactivity

The compatibility between KIR ligands on donor and recipient cells expressing HLA-B and HLA-C antigens has been proposed as a key parameter in evaluating NK cell allo-reactivity (14, 16). KIR-ligand compatibility has been shown to be a prognostic factor in the clinical outcome after HCT (17, 18), and can possibly be a key component in the prognosis of HCT from an HLA-C mismatched donor, particularly given the primary role of HLA-C as ligands for KIR.

In our study, we found no correlation between KIR-ligand compatibility and CTLp allo-reactivity. CTLp allo-reactivity was detected in 44 of the 90 (49%) donor–recipient pairs that were KIR-ligand compatible and similarly in 12 of the 25 (48%) donor–recipient pairs that were KIR-ligand incompatible (*p* = 1, Fisher’s exact test). MFI values of the recipient and of the donor mismatched HLA-C antigens were not associated with KIR-ligand compatibility/incompatibility between the donor and the recipient (recipient median MFI 164 and 180 in KIR-ligand compatible and KIR-ligand incompatible, respectively, *p* = 0.424, MWU test; donor median MFI 164 in both cases). Thus, we did not find any relevance of donor–recipient KIR-ligand compatibility in the association between HLA-C antigen MFI levels and CTLp results.

### Univariate logistic regression analysis

Table 1 describes the results of a univariate logistic regression analysis to test whether CTLp outcome can be predicted by the studied variables. The recipient’s mismatched HLA-C antigen cell surface expression level as expressed in MFI units was predictive of CTLp outcome, both when calculated as a discrete numeric or as a binary variable. Donor HLA-C antigen cell surface expression level almost reached statistical significance for prediction of CTLp outcome when calculated as a discrete numeric variable, and reached significance when considered as a binary variable based on alleles of extreme high- (MFI > 200) and low- (MFI ≤ 115) expression levels. Furthermore, we found that the interaction variable product of the HLA-C antigen MFI levels of the donor and recipient is significantly predictive of CTLp result. CTLp is 10-fold more likely to be negative in cases where both HLA-C mismatched alleles are of low expression.

**Table 1:** **Univariate logistic regression analysis for prediction of CTLp outcome**.

	Odds ratio	95% confidence interval	*p*
**DISCRETE NUMERICAL VARIABLES (*n* = 115)**			
Patient mismatched HLA-C MFI	1.759	1.199–2.580	0.004
Donor mismatched HLA-C MFI	1.384	0.987–1.939	0.059
Interaction variable: recipient × donor HLA-C MFI	1.002	1.001–1.003	0.008
Shared HLA-C allele MFI	1.012	0.657–1.559	0.957
**BINARY VARIABLES**			
Patient mismatched HLA-C MFI (*n* = 68)	2.947	1.077–8.066	0.035
Donor mismatched HLA-C MFI (*n* = 74)	2.923	1.126–7.585	0.027
Combined: recipient and donor HLA-C MFI (*n* = 41)	10.400	1.928–56.102	0.006
Shared HLA-C allele MFI (*n* = 75)	1.277	0.354–4.613	0.709
Amino-acid position-116 mismatch (*n* = 115)	1,176	0.537–2.577	0.685
KIR-ligand incompatibility (*n* = 115)	0.965	0.398–2.343	0.937

*MFI was calculated as discrete numerical variable, as described by Apps et al. (1) or dichotomized as a binary variable based on the extreme high- and low-expression mismatches. In regard to the discrete parameters, the change in OR shown for HLA-C expression represents an increase of 50 MFI expression units*.

### Multivariate logistic regression analysis

Table 2 describes the results of a multivariate logistic regression analysis to test whether CTLp outcome can be predicted by the studied variables. In a model based on discrete numerical variables, we found that the cell surface expression level of the recipient and donor mismatched HLA-C alleles, both separately and in combination, are independent predictors of CTLp result. In a model of binary variables only the combined donor–recipient parameter was predictive after stepwise elimination.

**Table 2:** **Multivariate logistic regression analysis for prediction of CTLp outcome**.

	Odds ratio	95% confidence interval	*p*
**MODEL I: DISCRETE NUMERICAL VARIABLES (*n* = 115)**			
Patient mismatched HLA-C MFI	7.889	1.987–31.324	0.003
Donor mismatched HLA-C MFI	6.339	1.594–25.202	0.009
Interaction variable: recipient × donor HLA-C MFI	0.991	0.984–0.999	0.020
**MODEL II: BINARY VARIABLE (*n* = 41)**			
Combined: recipient and donor HLA-C MFI	10.400	1.928–56.102	0.006

*MFI was calculated as discrete numerical variable, as described by Apps et al. (1) or dichotomized as a binary variable based on the extreme high- and low-expression cases. In regard to the discrete model, the change in OR shown for HLA-C expression represents an increase of 50 MFI expression units*.

## Discussion

### Rationale for the study

Multiple publications have reported that the CTLp frequency assay is able to predict clinical outcome of HCT (7, 19–21), which is the reason why this assay is incorporated in the clinical decision making of donor selection in several Dutch transplant centers (6). The CTLp assay quantifies the frequency of donor CTLp’s capable of responding to mismatched HLA Class I antigens presented on the recipient’s cells. The CTLp response against non-self HLA Class I antigens is dependent on physical engagement between the allo-antigen and the cytotoxic T-cell (22), although the exact mode of recognition is not yet understood to a degree that would allow prediction of CTLp allo-reactivity (23). Clearly, the presence of a mismatched Class I antigen on the surface of the target cell is essential for a positive CTLp response.

Cell surface expression of HLA-C antigens is regulated by interplay of genetic and epi-genetic factors (24, 25). Differential expression of HLA-C is notably associated with clinical ramifications such as HIV control and risk of CD (1, 10). Recently, Apps et al. (1) determined the mean expression levels for each two-digit HLA-C allotype and showed a direct genetic influence of HLA-C antigen expression level on the course of HIV infection. The authors demonstrated that HIV peptides presented by highly expressed HLA-C alleles were more likely to elicit CTL responses in comparison to low-expressed HLA-C allotypes even when presenting the same peptides. These results suggest that increased HLA-C expression can intensify a CTL-mediated immune response, consequently leading to enhanced protection in HIV infection.

The rationale for the current study was to investigate whether cell surface expression levels of HLA-C alleles can assist in predicting CTLp allo-reactivity, as a component of the donor selection process for patients who do not have a fully HLA-matched donor.

### Recipient HLA-C expression level

In the current study, we found that CTLp allo-reactivity is associated with the cell surface expression of the mismatched HLA-C antigens of the recipient. The presence of HLA-C antigens that are highly expressed on the surface of recipient cells was significantly correlated with a higher frequency of alloreactive CTLp. Moreover, logistic regression analysis showed that recipient HLA-C antigen MFI is a significant predictor of positive CTLp. This result is in agreement with the preceding publications reporting stronger CTL responses in the context of high-HLA antigen expression (26, 27) in HIV patients. Our study is the first to report the association between CTLp assay outcome and the genetic predisposition for HLA-C cell surface expression levels in HCT recipient–donor pairs.

Our findings are significant at a population level analysis of a heterogeneous group of 115 patient–donor pairs in spite of partial overlap between the MFI ranges of the studied groups. Thus, we assume that the significance of CTLp and antigen expression associations will be even more pronounced in our future research, upon prospective analysis of both the expression of HLA-C and CTLp frequency in specific patients and in their potential prospective donors.

### Donor–recipient matching at residue-116 in the HLA-C antigen

Several publications have shown that a mismatch between donor and recipient at amino-acid position-116 of the HLA-C protein is associated with inferior clinical outcome in HCT, namely, higher incidence of GvHD (4, 15, 28). Our group has demonstrated that CTLp assay outcome is also associated with donor–recipient matching at position-116 (11, 23). Residue-116 is located in the F-pocket of the peptide binding groove of the HLA Class I molecule (22). The amino-acid at position-116 does not interact directly with the TCR, but rather, affects peptide binding to the HLA molecule. Therefore, the pronounced allo-immunogenicity of a mismatch at position-116 is likely to be due to presentation of foreign peptides to the TCR.

In the current study, the association between CTLp result and recipient HLA-C cell surface expression level was more pronounced in donor–recipient pairs that are mismatched for the amino-acid at position-116 of the HLA-C antigen. The current findings demonstrate that allo-reactivity as expressed and measured by CTLp frequency is multi-factorial. CTLp responsiveness is determined by the allotype of the recipient’s HLA-C antigen together with the identity of the peptide presented in the peptide binding groove of the allo-antigen.

Donor–recipient KIR-ligand compatibility did not affect the association between CTLp outcome and HLA-C expression levels. This finding confirms the conclusions of previous publications that found of no association between KIR-ligand compatibility and CTLp allo-reactivity (11, 23).

### Donor HLA-C expression level

We also identified an association between the HLA-C cell surface expression level on the donor cells and the CTLp assay result in donor–recipient pairs where the recipient’s mismatched HLA-C antigen expression is low. The donor HLA-C MFI was a significant variable for prediction of CTLp outcome in logistic regression analysis, both independently and as an interaction variable together with the recipient MFI. Furthermore, we found that when both donor and recipient carry a mismatched HLA-C allele of low-cell surface expression, the likelihood of a negative CTLp result is 10-fold more likely than in a situation where one of the mismatched alleles is of high expression.

This seems surprising, as donor HLA antigens do not directly participate in the CTL response against mismatched recipient antigens. We hypothesize that the association between the donor HLA-C cell surface expression level and the CTLp allo-response is a result of differences in thymic T-cell selection and its impact on the TCR repertoire on donor cells. This hypothesis is in line with previous findings by Zemmour et al. (29), who also suggested that the HLA-C antigen trait of low expression has a unique impact on thymic T-cell selection. Also, more recent publications have pointed at the possible relationship between the cell surface expression level of HLA-C antigens and thymic T-cell selection and the consequent HIV viremia control by HLA-C restricted T-cells (30–32). Low expression of HLA-C antigens in the thymus may affect the positive and negative selection and as a consequence, influence the repertoire and TCR avidity of mature T-cells. This hypothesis remains to be confirmed.

### Summary and future directions

The present study shows that there is a significant association between cell surface expression levels of HLA-C alleles and CTLp allo-reactivity. Based on their genetically predisposed expression levels, both recipient and donor mismatched HLA-C alleles can contribute to prediction of GvH allo-reactivity. Prediction of allo-reactivity is of importance for the selection of optimal HLA mismatched donors for patients in need of HCT who do not have a matched donor. Future studies will aim at the validation of our findings with clinical data of HCT in order to establish the usefulness of incorporating HLA-C expression levels together with CTLp results for prediction of GvH allo-reactivity.

## Conflict of Interest Statement

The authors declare that the research was conducted in the absence of any commercial or financial relationships that could be construed as a potential conflict of interest.
